# Retrograde cannulation of femoral artery: A novel experimental design for precise elicitation of vasosensory reflexes in anesthetized rats

**DOI:** 10.1016/j.mex.2020.101017

**Published:** 2020-07-29

**Authors:** Ravindran Revand, Sanjeev K. Singh

**Affiliations:** Department of Physiology, Institute of Medical Sciences, Banaras Hindu University, Varanasi 221005, UP, India

**Keywords:** Vasosensory reflexes, Perivascular afferents, Nociception, Local vascular bed, Femoral arterial cannulation

## Abstract

Vasosensory reflex responses are elicited by instillation of nociceptive agents in a segment of peripheral blood vessel. A novel method for the stimulation of perivascular afferents was designed by retrograde cannulation of femoral artery, using a 24G, double ported polyethylene cannula. The vertical port of which was used to inject the algogen into the artery and horizontal port to measure the BP continuously, as this port was connected to the pressure transducer. Previously, separate carotid artery cannulation was used for the BP recording. But our experimental design excluded the need for carotid artery cannulation that might compromise the circulation to the CNS centers mediating cardiorespiratory reflex responses. After cannulation, the proximal end of femoral artery became an end artery and the drugs were instilled retrogradely. The volume of chemicals was kept minimal (100 µl) and the ipsilateral femoral vein was also ligated. These measures made sure that the instilled drug remained in a local segment of femoral artery and did not spill out to the systemic circulation. Further, there was no increase in the water content of ipsilateral paw as compared to the contralateral paw. This finding also substantiates our proposition regarding minimal systemic spillage.

• The femoral artery is cannulated by a double ported cannula.
• This cannula helps instillation of algogen and BP measurement simultaneously.
• Retrograde instillation helps to deposit the algogen in a local segment of femoral artery.

## Graphical abstract

**Figure:**
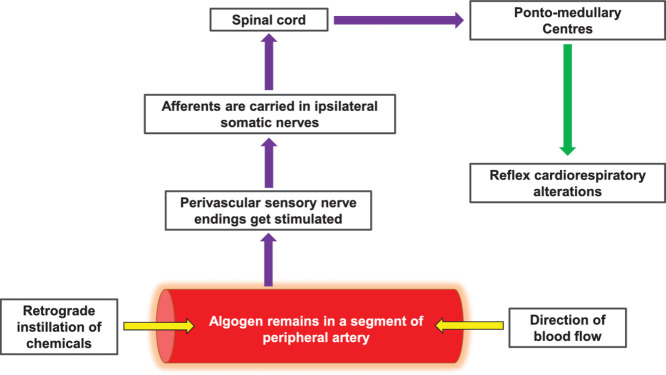

**Specifications Table**

**Table:** 

Subject Area:	Medicine and Dentistry
More specific subject area:	*Vasosensory reflexes*
Method name:	*Retrograde instillation of algogens in femoral artery: A novel approach*

## Method details

### Overview

**Fig. 1:**
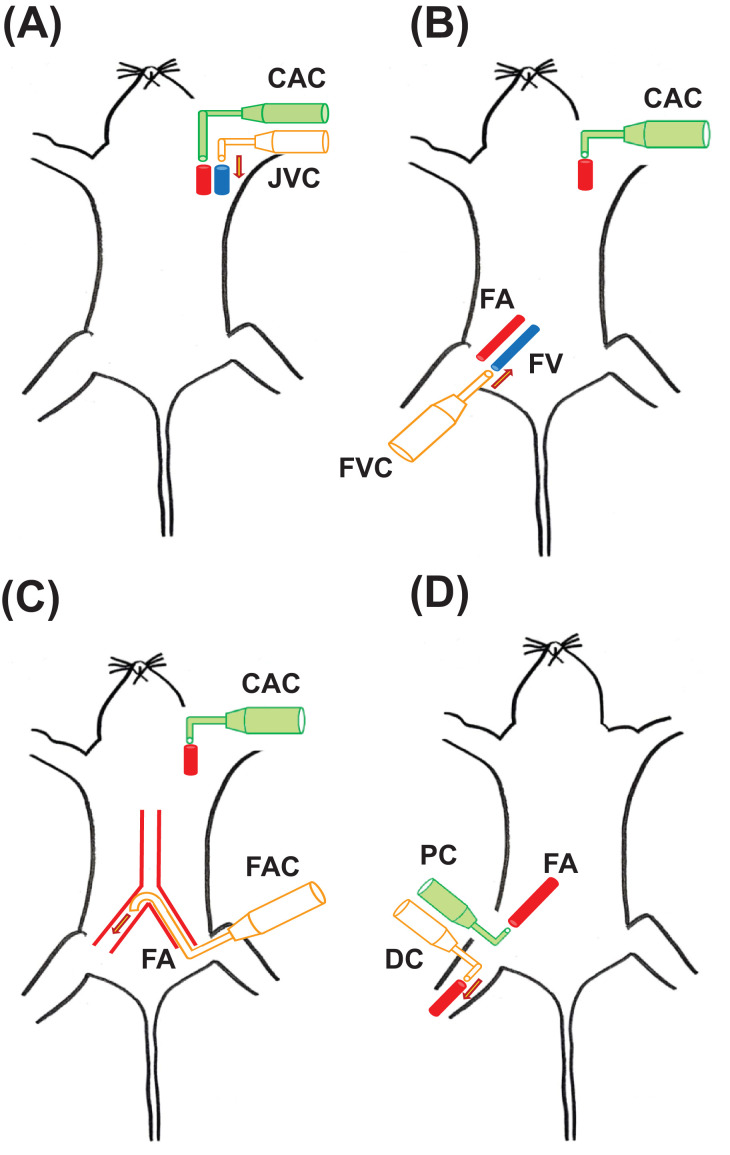
Schematic diagrams showing earlier experimental designs to study vascular reflexes in rats. (A) Jugular vein cannula (JVC) is utilized to inject drugs and carotid artery cannula (CAC) is connected to a pressure transducer for blood pressure (BP) recording. (B) Femoral vein cannula (FVC) is utilized to inject drugs and CAC to record BP. (C) A femoral artery cannula (FAC) is advanced retrogradely from the left femoral artery till the level of abdominal aorta bifurcation and drugs are injected into the right femoral artery anterogradely. CAC is utilized for BP recording. (D) Dual cannulation of the right femoral artery is performed. The proximal cannula (PC) is connected to pressure transducer to measure BP while the distal cannula (DC) serves to inject the drug in a local segment of an artery. The arrows in the diagrams represent the direction of injection of drugs. FA = femoral artery; FV = femoral vein.

Medium and small sized peripheral blood vessels, both arteries and veins are innervated by large number of peptidergic neurons [1]. These small diameter myelinated Aδ and unmyelinated C fibers are known to play a major role in nociception. In animal models, the perivascular sensory terminals have been shown to become activated on exposure to inflammatory mediators, toxins, chemical agonists etc. in the circulation and convey the information of local tissue damage to the CNS. The CNS then modulates cardio-respiratory (CVR) parameters via reflex mechanisms [[2], [3]–4]. Such reflex CVR responses elicited by nociceptive agents present in the blood vessels are known as ‘vasosensory reflex responses’. Many workers have demonstrated different experimental designs to elicit these reflex responses which are briefly described in this article, just for the understanding of our present experimental design (Fig. 1).

In earlier experimental design, the nociceptive agents when injected into the jugular vein, could have reached the chambers of the heart, roots of great vessels and further to pulmonary circulation, thereby stimulating the cardiopulmonary receptors directly, instead of stimulating perivascular sensory nerve terminals located in the walls of peripheral blood vessels (Fig. 1A). Jugular vein cannot be considered as a peripheral blood vessel because of its close proximity to the heart. Therefore, to study the role of peripheral blood vessels in elicitation of vasosensory reflexes, cannulation of femoral vein was performed (Fig. 1B) and similar responses of CVR parameters were reported [7]. Perivascular afferents could be more precisely activated if the nociceptive agonist is deposited in an artery than in a vein as VR1 is abundant in this region. Another drawback of femoral vein cannulation was that the injected chemical could still reach the systemic circulation and stimulate the nociceptors located in the cardiopulmonary areas, muscles and joints.

In works elsewhere, a cannula was inserted into the left femoral artery and advanced retrogradely till the level of bifurcation of abdominal aorta. The agonist (bradykinin) was then injected into the right femoral artery anterogradely and vasosensory reflex responses were demonstrated [4]. In these experiments, a separate carotid artery cannulation was performed for BP recording (Fig. 1C). Carotid artery cannulation may significantly compromise the circulation of ponto-medullary centers which are the principal reflexogenic areas for regulation of CVR parameters. Earlier works from our laboratory, demonstrated vasosensory reflex responses by dual cannulation of the same femoral artery [2,3]. One cannula was placed proximally and connected to BP transducer, while the other cannula was inserted distally to inject the nociceptive agent towards the ipsilateral paw (Fig. 1D). This design helped to avoid the cannulation of carotid artery, but there was a significant increase in the water content of the ipsilateral paw. This suggested that the injected drug reached the capillary microvasculature of the paw and caused edema by increasing the local vascular permeability. There is also a possibility that the drugs might have crossed the capillary bed and have reached the systemic circulation. This could directly stimulate the free nerve endings and cardiopulmonary receptors.

## Retrograde instillation of algogens in femoral artery: A novel approach

Animal experiments were performed after obtaining approval from the Animal Ethical Committee of the Institute of Medical Sciences, Banaras Hindu University, Varanasi (Ref. No. Dean/2019/IAEC/1627 dated 17.11.2019). The animals were handled in accordance with the EU directive 2010/63/EU.

### Drugs, solutions and instruments

Urethane (Product No. U2500) was procured from Merck, Germany. The salt was stored in a dark dry place at room temperature (28 ± 2 °C). Anesthetic solution was prepared by dissolving urethane salt in double distilled water in the concentration of 0.5 g/ml. Heparin sulphate (1000 IU/ml) was obtained from Biological Evans Ltd., India. Before each experiment, heparinized saline was freshly prepared by mixing 1.0 ml of heparin sulphate in 50 ml of 0.9% saline. Histamine dihydrochloride salt (Product No. H7250) was procured from Sigma Chemicals Company, MO, USA and was kept in refrigerator at 2–8 °C. Stock solution (1000 mM) of histamine was prepared in distilled water and stored in refrigerator (2–8 °C). Subsequent dilutions of histamine (10 mM) were prepared using 0.9% saline prior to each experiment. Castroviejo corneal scissors, 4.5” curved blade (Model No. 4336853428) were procured from Kretzer, Germany. Optivisor^Ⓡ^ magnifying head loop (Model No. 633096000519) was bought from Donegan Optical Company, USA. The BP transducer, force transducer, ECG needle electrodes, data acquisition system and bridge amplifier were all established and standardized by Power Lab 26T, AD Instruments^Ⓡ^, Australia.

### Animals and anesthesia

Healthy adult male albino rats (Charles-Foster strain; 202.50 ± 12.50 g) were housed at 12:12 h light/dark cycle at 28 ± 2 °C and food/water was provided *ad libitum*. Freshly prepared urethane solution was injected intraperitoneally (1.5 g/kg) in the left lower abdomen 1 cm lateral to the midline. The animals were then allowed to remain in a calm and dark environment. Periodic assessment of the level of anesthesia was done at every 15 min intervals. An additional dose (50–100 mg) of urethane was given, if required. The level of anesthesia was assessed by eliciting corneal reflex using a cotton wisp and withdrawal reflex to a painful stimulus, by squeezing the paw gently with blunt forceps. Absence of corneal reflex and withdrawal reflex was considered as adequate level of anesthesia.

### Tracheostomy

**Fig. 2:**
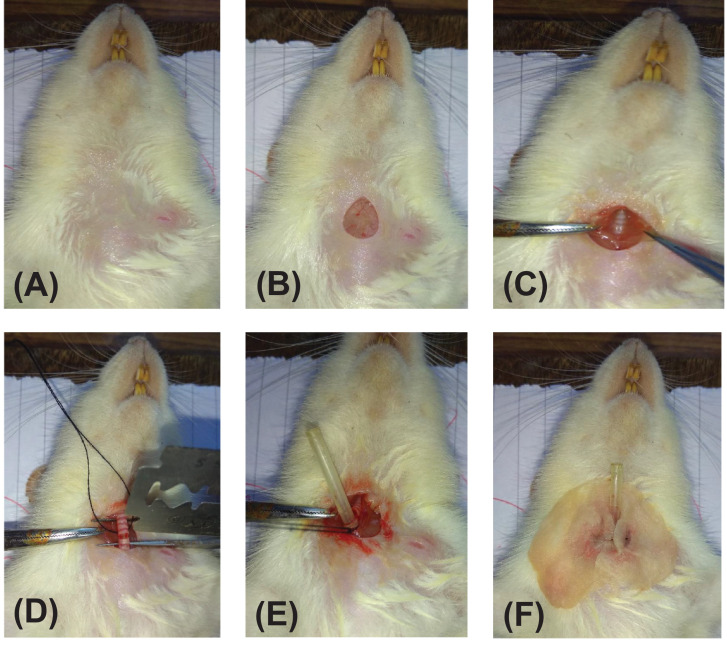
Photographs demonstrating the steps of tracheostomy in rats. (A) Anterior aspect of neck is shaved. (B) Midline neck incision is made and cervical fascia is exposed. (C) Strap muscles are separated laterally to expose the trachea. (D) A thread is passed under the trachea and a small transverse nick is made between the tracheal rings using a blade. (E) A polyethylene tube is inserted into the trachea and secured tightly by the thread. (F) The dissection site is covered with cotton moistened with normal saline.

Under the effect of anesthesia, the skin over the anterior aspect of neck was shaved (Fig. 2A) and a small midline incision was made to expose the deep cervical fascia (Fig. 2B).The thyroid gland was identified, isolated and retracted laterally. The strap muscles of the anterior neck were then separated along the midline and the trachea was exposed (Fig. 2C). A thread was passed beneath the trachea and a small transverse nick was made between the 5th and 6th tracheal rings (Fig. 2D). A polyethylene tube of appropriate size was inserted into the trachea and secured tightly by the thread (Fig. 2E). The length of the tube was kept minimal to avoid the dead space. The dissection site was finally covered with a piece of cotton which was periodically moistened by normal saline (Fig. 2F). This prevented tissue damage due to drying of exposed tissues. Tracheal secretions were aspirated as and when needed. Under the effect of anesthesia, the tongue might fall back and obstruct the upper airway. Therefore, the tracheal cannulation was performed to reduce the respiratory distress to the animal.

### Dissection and cannulation

**Fig. 3:**
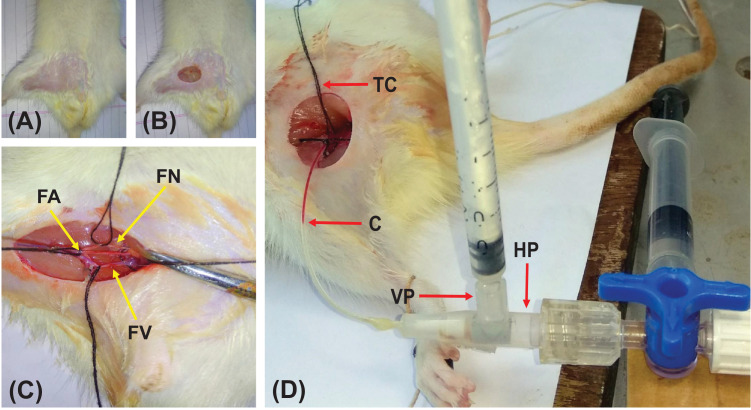
Photographs showing the steps of femoral artery cannulation in our experimental design. (A) The skin in the region of femoral triangle is shaved. (B) A small incision is made in the inguinal region to expose the superficial fascia. (C) Deeper dissection is done to skeletonize the femoral vein (FV), femoral artery (FA) and femoral nerve (FN) from medial to lateral. Threads are passed around these structures to retract them from each other and for manipulations. (D) The femoral artery is cannulated by a double ported polyethylene cannula retrogradely. TC is a thread clamp to regulate the flow of blood into the cannula. The fluctuating interface between the arterial blood and heparinized saline is marked as C. VP is the vertical port of the cannula for drug instillation into femoral artery which contains an injection valve and HP is the horizontal port connected to the pressure transducer for measuring the blood pressure.

The skin in the region of right femoral triangle was prepared and a small incision was made at the midpoint of an imaginary line joining the urethral orifice and the hip joint (Fig. 3A and B). Further dissection of the superficial fascia and extra-peritoneal pad of fat exposed the femoral vessels. The superficial epigastric artery was then ligated and the extra-peritoneal pad of fat was retracted superiorly. The femoral artery, femoral vein and femoral nerve were separated from each other with the help of glass seeker and threads were passed around each of them (Fig. 3C). Nearly 1 cm length of femoral artery was dissected and accessed. Then the femoral artery was clamped proximally using a thread as a clamp. A small nick was made on the femoral artery distal to the clamp using Castroviejo corneal scissors. A 24G, double ported polyethylene cannula, designed in our laboratory, filled with freshly prepared heparinized saline (20 IU/ml) was inserted into the femoral artery retrogradely. The heparinized saline prevented clotting of blood in the cannula and helped us to visualize the fluctuating blood column throughout the experiment (Fig. 3D).The cannula was secured tightly to the arterial wall by threads. The whole dissection and cannulation process was performed under 2.75× magnification using Optivisor^Ⓡ^ (Donegan optical company, USA). The femoral artery was chosen for cannulation in our experimental design because of its easy accessibility, comparatively larger caliber and tough nature of its wall.

### Description of the cannula

**Fig. 4:**
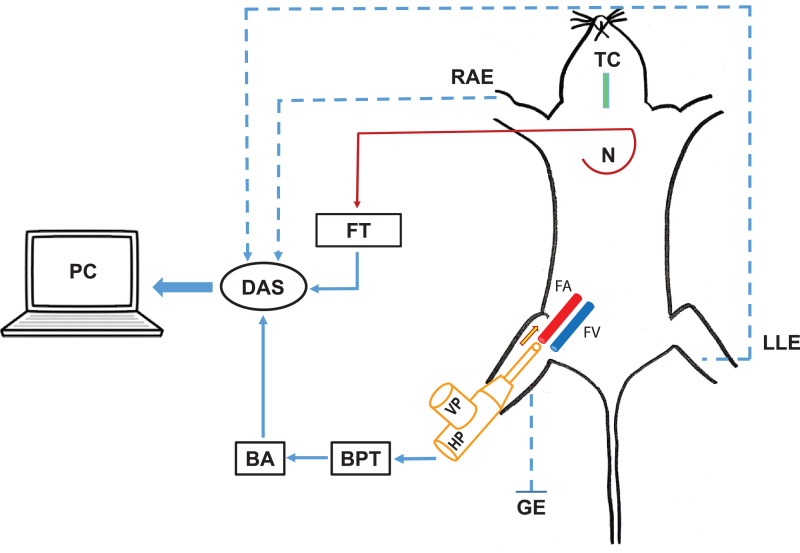
Schematic diagram depicting our experimental design for the elicitation of vasosensory reflex responses. Femoral artery is cannulated retrogradely by a double ported cannula. The vertical port (VP) of the cannula is utilized for instillation of drugs and the horizontal port (HP) is connected to a blood pressure transducer (BPT). This in turn is connected to a bridge amplifier (BA) and then to the data acquisition system (DAS) that gives the blood pressure on a personal computer (PC). TC is the tracheal cannula and N is a curved needle that is connected to the force transducer (FT) by means of a thread, to record respiratory excursions. RAE, LLE and GE are the right arm electrode, left leg electrode and the ground electrode respectively connected in standard limb lead-II configuration that provides electrocardiographic (ECG) recordings on the PC.

The double-ported cannula was prepared in our laboratory. The tip of the cannula was beveled to facilitate insertion into the femoral artery through the small nick. The cannula had two ports – a vertical and a horizontal port (Fig. 3D). The vertical port containing an injection valve was utilized for the instillation of chemicals/drugs and the horizontal port was used to record BP simultaneously, by connecting it to a pressure transducer (Fig. 4).

By using this double ported cannula, we were able to avoid the cannulation of carotid artery for BP measurement as it was done elsewhere [[4], [5], [6]–7]. This minimized the surgical trauma to the animal and prevented the circulatory compromise to the ponto-medullary centers. After cannulation, the femoral artery became an end artery and the volume of drugs was also kept minimal (100 µl) and constant. The drug was instilled retrogradely against the direction of blood flow in the femoral artery and the ipsilateral femoral vein was also ligated. These measures made sure that the instilled drug remained in a local segment of femoral artery just proximal to the cannula and spillage into the systemic circulation was least possible. Another major advantage of this experimental design is that the exact point of drug instillation could be ascertained, as it marks an artifact by interrupting the BP recording for a fraction of second.

### Recording of CVR parameters

After cannulation, the horizontal port of the cannula was connected to the BP transducer. This in turn was connected to a bridge amplifier and finally to a data acquisition system (DAS; Power Lab 26T, AD Instruments^Ⓡ^, Australia) which provided the BP recording on the computer screen (Fig. 4). The electrocardiographic (ECG) potentials were recorded using needle electrodes, connected in standard limb lead-II configuration. The respiratory movements were recorded by securing the skin over the xiphisternum using a curved needle and connecting it to a force transducer (AD Instruments^Ⓡ^, Australia) *via* a thread. The BP, respiratory excursions and ECG were recorded on a personal computer connected to the DAS (Power Lab 26T, AD Instruments^Ⓡ^, Australia). The respiratory frequency (RF), mean arterial pressure (MAP) and heart rate (HR) were obtained from the original recordings. At the end of experiments, a known volume (1 ml) of air was injected into the quiescent lung of sacrificed animal through the tracheal tube and the corresponding deflection was computed as ‘x’. The average amplitude of respiratory excursions for a period of 5 s was measured as ‘h’. For calculation of respiratory minute volume (RMV), the height (in mm) of respiration was converted to volume (in ml) by using the formula [h/x] and was multiplied with the RF.

Thus in our experiments, we elicited vasosensory reflexes by instilling nociceptive agent (histamine) in a local segment of femoral artery and studied the changes in the BP, HR, RF and RMV parameters.

## Method validation

### Estimation of water content of paws

At the end of experiments, both the hind paws were disarticulated at the level of ankle joint. The wet weight of paws was determined and then they were dried in an electric oven (90 °C) for a period of 72 h. The difference between wet weight and dry weight of a paw provided its water content and was expressed as percentage of wet weight. In our experiments with histamine, the water content in ipsilateral paw was not significantly different as compared to the contralateral side for all concentrations (1 mM, 10 mM and 100 mM) of histamine. This finding supports our proposition that the injected histamine is deposited in a local segment of femoral artery and did not spill out into the systemic circulation.

### Ipsilateral neurotomy attenuated histamine-induced CVR responses

**Fig. 5:**
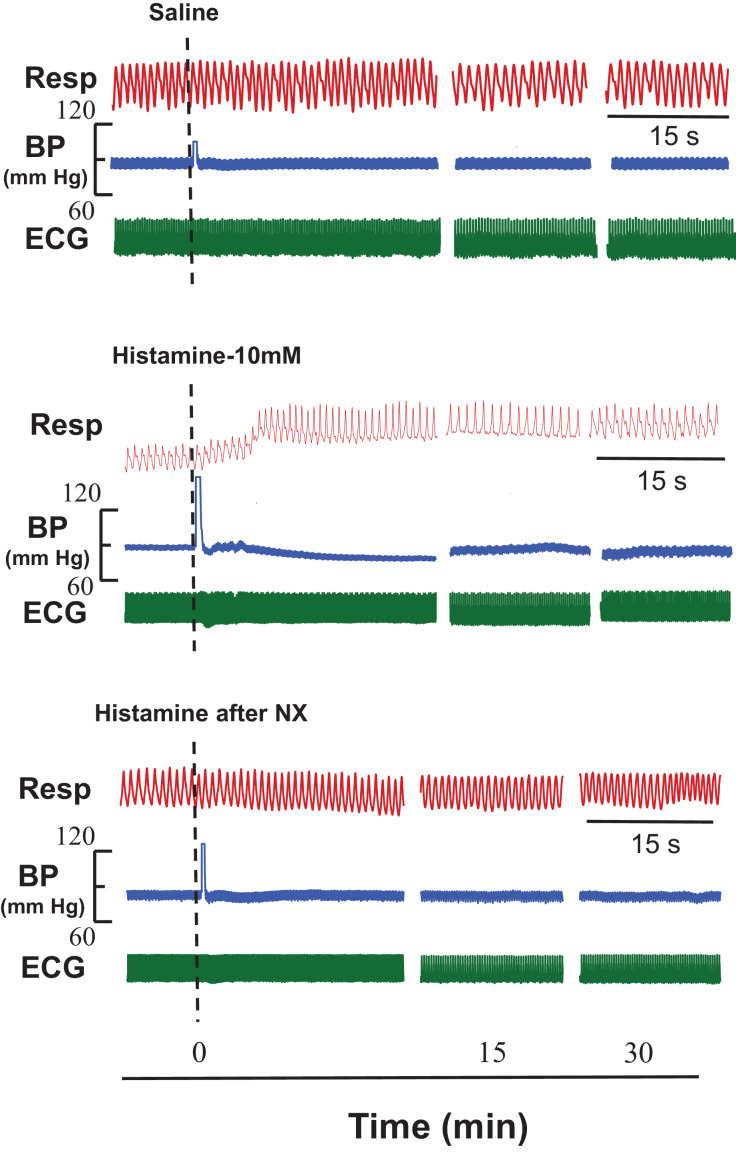
Original recordings showing the effect of intra-arterial (i.a) instillation of histamine/saline on respiration (Resp), blood pressure (BP) and electrocardiogram (ECG) in three different groups of rats (*n*=6). Instillation of saline did not produce significant changes in the cardiorespiratory (CVR) parameters as shown in upper panel while histamine (i.a; 10 mM) evoked tachypnoeic, hypotensive and bradycardiac responses as shown in middle panel. The lower panel shows the effects of ipsilateral neurotomy (NX) on histamine-induced responses. It can be clearly seen that ipsilateral somatic neurotomy blocked the histamine-induced CVR changes. Dotted lines indicate the point of injection of saline/histamine. The time scale is given in the lower panel.

Instillation of histamine elicited concentration dependent tachypnoeic, hypotensive and bradycardiac responses, while in control experiments equi-volume of saline did not, eliminating the probability of stretch/ischemia-induced responses on the blood vessel wall. The shorter latencies of the responses (2–7 s) favor the neuronal involvement in producing these responses. The histamine-induced reflex CVR responses were significantly attenuated after ipsilateral femoral and sciatic nerve sectioning (Fig. 5). The above findings also support our hypothesis that these reflex responses arise from the local vascular bed and their afferents run in the ipsilateral somatic nerves.

## Conclusion

In conclusion, this novel experimental design provides an opportunity to understand the reflexes originating from a local vascular bed leading to alteration in the CVR parameters. Further, it also allows us to record all the CVR parameters with minimal possible surgical interventions, thereby avoiding unnecessary tissue damage or compromise to the vital centers.

## Appendix Supplementary materials

Image, video 1
